# On fractional order models for Hepatitis C

**DOI:** 10.1186/1753-4631-4-1

**Published:** 2010-03-18

**Authors:** E Ahmed, H A El-Saka

**Affiliations:** 1 Mathematics Department, Faculty of Science, Mansoura University, 35516, Mansoura, Egypt; 2 Mathematics Department, Faculty of Science, Mansoura University, 34517, New Damietta, Egypt

## Abstract

In this paper we present a fractional order generalization of Perelson et al. basic hepatitis C virus (HCV) model including an immune response term. We argue that fractional order equations are more suitable than integer order ones in modeling complex systems which include biological systems. The model is presented and discussed. Also we argue that the added immune response term represents some basic properties of the immune system and that it should be included to study longer term behavior of the disease.

## 1. Introduction

Hepatitis C is one of many serious infectious diseases affecting humans. An estimated about 170 million people worldwide are infected with hepatitis C. No vaccine against hepatitis C is available. Egypt has the highest rate of infections by hepatitis C virus (HCV) genotype 4. Chronic HCV infection is the main cause leading to liver transplant or death [1]. Antiviral therapy has been used to treat chronically HCV infected patients. Relatively successful treatment contains pegylated interferon (PEG-IFN) and ribavirin (RBV). Typical response ate biphasic beginning with rapid viral decline phase followed by slower decline phase. Sometimes there is more complicated behavior but they will not be discussed here. Extensive and impressive studies have been done by Perelson and his group [2-5]. Here we present a fractional order generalization of a basic HCV model [2] including the immune response (Ir) term proposed in [3].

In sec. 2 we argue that fractional order equations are more suitable than integer order ones in modeling complex systems which include biological systems. In sec.3 the model is presented and discussed. Also we argue that the added immune response term represents some basic properties of the immune system and that it should be included to study longer term behavior of the disease.

## 2. Fractional Equations

Caputo's definition for derivative of order 0 < α ≤ 1 is given by [6]

where Γ (α) is the Gamma function and *f*'(*s*) is the first derivative. It is known that FO is more suitable in describing complex adaptive systems e.g. biological systems since they naturally represent fractal, memory and non-locality effects [7-9].

## 3. The model and conclusions

The fractional order hepatitis C virus (HCV) model is given by(1)

Where 0 < α ≤ 1, *T *represents uninfected hepatocytes, *I *represents infected hepatocytes and *V *represents virus. The model assumes that uninfected hepatocytes are produced at a constant rate *s*, die at rate, *d*, per cell and are infected at constant rate β. Infected hepatocytes are lost at a rate δ per cell. Viral particles (virions) are produced at rate *p *per infected hepatocyte and cleared at rate *c *per virion. Chronic HCV infection is treated using interferon-a in combination with the antiviral drug ribavirin. Interferon-a acts primarily by blocking the production/release of new virus, although we also allow for a treatment effect in blocking *de novo *infection. The efficacy of treatment in blocking virion production and reducing new infections are described by two parameters, ε_*p *_and η, respectively. For example, a treatment efficacy in blocking virion production of 95% corresponds to ε_*p *_= 0.95.

**Figure 1:**
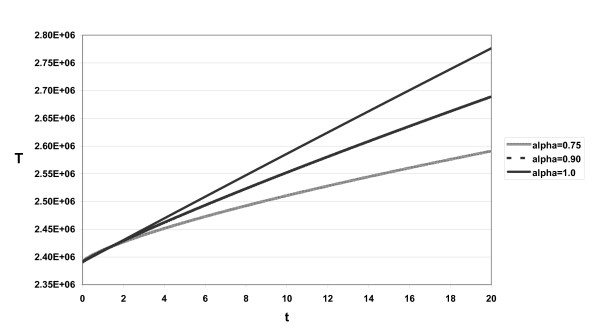


**Figure 2:**
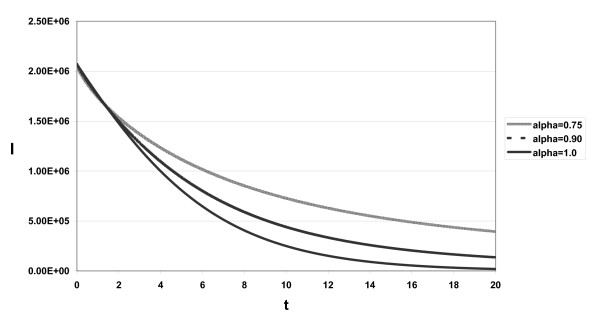


**Figure 3:**
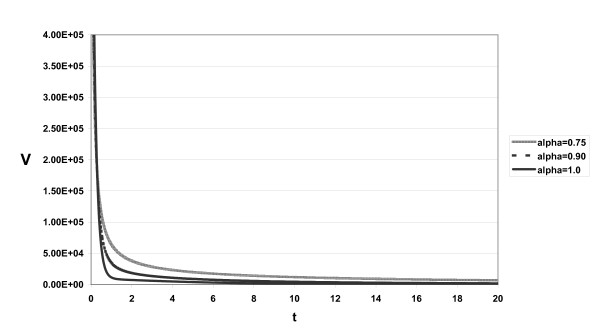


The approximate solutions displayed in Figs. 1, 2, 3 for different α and *s *= 2.6 × 10^4^, *d *= 0.0026, η = 0.95, β = 2.25 × 10^-7^, δ = 0.26, *c*_2 _= 5 × 10^6^, *c *= 6.0, ε_*p *_= 0.99, *p *= 2.9

We have the following conclusions:

i) Mathematical models, when done by a group of mathematicians and HCV specialists give relevant results e.g. explaining the role of RBV [4], explaining the observed two phase decline hence deriving the role of the drug in initially blocking the viral secretion more than blocking the cells' infection etc. [4].

ii) The term added in equation (1) represents the well known high and low zone tolerance of the immune response (There is no Ir if the antigen number is either too high or too low [10]) since it vanishes for both limits *I *→ 0 and *I *→ *c*_2_. Immune response is significant for long time virus dynamics.

iii) Fractional order results show the realistic biphasic decline behavior of HCV but at a slower rate.

iv) Since multi-drugs are strongly recommended to avoid drug resistance it is proposed that a further drug should be used in addition to Peg-IFN and RBV. Notice that RBV may not be considered as independent drug since, when used alone, it has no effect on HCV.
